# Determining the Fate of Neurons in SCA3: ATX3, a Rising Decision Maker in Response to DNA Stresses and Beyond

**DOI:** 10.3389/fcell.2020.619911

**Published:** 2020-12-23

**Authors:** Yingfeng Tu, Xiaoling Li, Xuefei Zhu, Xiaokang Liu, Caixia Guo, Da Jia, Tie-Shan Tang

**Affiliations:** ^1^Key Laboratory of Birth Defects and Related Diseases of Women and Children, Department of Paediatrics, West China Second University Hospital, State Key Laboratory of Biotherapy and Collaborative Innovation Center of Biotherapy, Sichuan University, Chengdu, China; ^2^Hebei Key Laboratory of Applied Chemistry, School of Environmental and Chemical Engineering, Yanshan University, Qinhuangdao, China; ^3^Guangdong Key Laboratory for Genome Stability & Disease Prevention, Shenzhen University Health Science Center, Shenzhen, China; ^4^Beijing Institute of Genomics (China National Center for Bioinformation), University of Chinese Academy of Sciences, Chinese Academy of Sciences, Beijing, China; ^5^State Key Laboratory of Membrane Biology, Institute of Zoology, University of Chinese Academy of Sciences, Chinese Academy of Sciences, Beijing, China; ^6^Institute for Stem Cell and Regeneration, Chinese Academy of Sciences, Beijing, China

**Keywords:** neurodegenerative diseases, spinocerebellar ataxia type 3, ataxin-3, DNA damage response, apoptosis

## Abstract

DNA damage response (DDR) and apoptosis are reported to be involved in the pathogenesis of many neurodegenerative diseases including polyglutamine (polyQ) disorders, such as Spinocerebellar ataxia type 3 (SCA3) and Huntington's disease (HD). Consistently, an increasing body of studies provide compelling evidence for the crucial roles of ATX3, whose polyQ expansion is defined as the cause of SCA3, in the maintenance of genome integrity and regulation of apoptosis. The polyQ expansion in ATX3 seems to affect its physiological functions in these distinct pathways. These advances have expanded our understanding of the relationship between ATX3's cellular functions and the underlying molecular mechanism of SCA3. Interestingly, dysregulated DDR pathways also contribute to the pathogenesis of other neurodegenerative disorder such as HD, which presents a common molecular mechanism yet distinct in detail among different diseases. In this review, we provide a comprehensive overview of the current studies about the physiological roles of ATX3 in DDR and related apoptosis, highlighting the crosslinks between these impaired pathways and the pathogenesis of SCA3. Moreover, whether these mechanisms are shared in other neurodegenerative diseases are analyzed. Finally, the preclinical studies targeting DDR and related apoptosis for treatment of polyQ disorders including SCA3 and HD are also summarized and discussed.

## Introduction

Dominant inheritance of mutant Ataxin-3 (ATX3) leads to neurodegenerative disorder Machado-Joseph disease (MJD1, also known as spinocerebellar ataxia type 3/SCA3), with abnormal expansion of its C terminal polyglutamine (polyQ) repeats up to 55–87 in comparison to 10–51 in healthy individuals. The polyQ expansion length correlates positively with the disease severity and inversely with the age of disease onset (Kawaguchi et al., 1994; Riess et al., 2008; Matos et al., 2011; Lee et al., 2015). SCA3 is the most common form of spinocerebellar ataxia worldwide (Schols et al., 2004; Paulson, 2012), characterized by progressive ataxia, spasticity, and ocular movement abnormalities (Matos et al., 2011). The cytologic abnormalities of SCA3 is typically neuronal loss, to date as reported. Although the pathogenic ATX3 is expressed ubiquitously in various tissues and cell types, the mutation of this protein seems only to induce neuronal dysfunction. Especially, the neuronal loss selectively occurs in specific brain domains including cerebellum, substantia nigra, and striatum, suggesting a region-specific toxic mechanism. The wealth of information has provided deep insights into the physiological functions of ATX3 and the etiology of SCA3. Although the precise molecular mechanism underlying SCA3 pathogenesis remains enigmatic, a better understanding might be developed when we take recently-established functions of ATX3 in DNA damage response (DDR) and apoptosis into account.

The evolutionally conserved DDR network guarantees genome integrity upon various kinds of damage insults, which can be spontaneous, such as reactive oxygen species (ROS) derived from normal metabolism, or be exogenous such as ultraviolet (UV) from sunlight (Hoeijmakers, 2009; Ciccia and Elledge, 2010). DDR involves sophisticated signaling networks, with the ability to sense DNA damage, to transduce the signal and in the end to evoke cellular responses including DNA repair, DNA damage checkpoint, chromatin remodeling and apoptosis, contributing to both parental survival and faithful transmission of genetic information to offsprings (Friedberg, 2003; Harper and Elledge, 2007; Jackson and Bartek, 2009). DDR defects are reported to associate with various genetic diseases accompanied by neurodegeneration, such as AT (ataxia telangiectasia), Xeroderma pigmentosum, Trichothiodystrophy, and Cockayne syndrome (Friedberg et al., 1995; Ciccia and Elledge, 2010). Recently, ATX3 has been reported to exert crucial roles in DDR via interacting with various DDR proteins such as polynucleotide kinase 3'-phosphatase (PNKP), mediator of DNA damage checkpoint protein 1 (MDC1), checkpoint kinase 1 (Chk1), Huntingtin (HTT), Ku70, DNA-PKcs, 53BP1 and p97 (Chatterjee et al., 2015; Gao et al., 2015, 2019; Pfeiffer et al., 2017; Tu et al., 2017; Singh et al., 2019; Chakraborty et al., 2020). Aberrant polyQ expansion in ATX3 results in accumulation of DNA damage, activation of pro-apoptotic signaling pathway, and neurodegeneration in SCA3. In addition, abnormal polyQ expansion abrogates the expression of superoxide dismutase 2 (SOD2) (Araujo et al., 2011), which is involved in the clearance of ROS. Given the high level of oxygen consumption in nervous system, the ROS-induced cytotoxicity and oxidative DNA damage are believed to contribute to SCA3 pathogenesis. Similar to SCA3, Huntington's disease (HD), an autosomal dominant neurodegenerative disease, is also caused by polyQ expansion in the HTT protein (Ross et al., 2014). Consistently, many studies indicate the involvement of HTT in DDR and apoptosis (Zeitlin et al., 1995; Dragatsis et al., 2000; Kegel et al., 2000; Rigamonti et al., 2000; Leavitt et al., 2001; Anne et al., 2007; Maiuri et al., 2017), and there is accumulated DNA damage in HD patient samples and HD models (Browne et al., 1997; Bogdanov et al., 2001; Chen et al., 2007; Acevedo-Torres et al., 2008; Illuzzi et al., 2009; Enokido et al., 2010; Stack et al., 2010; Ferlazzo et al., 2014), indicating that abnormal DDR may play a general role in the pathogenesis of polyQ-related neurodegenerative diseases.

Apoptosis plays essential roles in organism development as well as in tumor-suppression, whose dysfunction closely relates to disease pathogenesis. As a well-established apoptosis regulator, p53 is involved in the pathogenesis of neurodegenerative diseases such as Alzheimer's diseases (AD), Parkinson's disease (PD), and HD (Chang et al., 2012). The important roles of p53 in the development of neurodegenerative diseases are associated with its interaction with various factors which are capable of promoting the progression of these diseases. A recent work has identified ATX3 as a novel deubiquitinating enzyme of p53. Normal ATX3 regulates the stabilization and pro-apoptotic function of p53, while polyQ expansion impedes the dissociation between ATX3 and p53, therefore enhances the stability and pro-apoptotic function of p53, supporting the involvement of p53 in SCA3 pathology (Liu et al., 2016). The antiapoptotic role of HTT and the observation that mutant HTT induces apoptosis (Zeitlin et al., 1995; Cooper et al., 1998; Hackam et al., 1998; Lunkes and Mandel, 1998; Reddy et al., 1998; Hodgson et al., 1999; Dragatsis et al., 2000; Rigamonti et al., 2000; Leavitt et al., 2001) suggest that apoptosis may also be another common pathway shared in polyQ-related neurodegenerative diseases.

In this review, we will summarize the recent advances concerning the roles of ATX3 in DDR and apoptosis, and emphasize the potential link of abnormal DDR and apoptosis to pathogenesis of SCA3 and other neurodegenerative diseases including HD. Finally, we describe preclinical studies targeting these essential pathways for treatment of neurodegenerative diseases including SCA3 and HD.

## The Deubiquitinase Activity of ATX3

**Figure 1:**
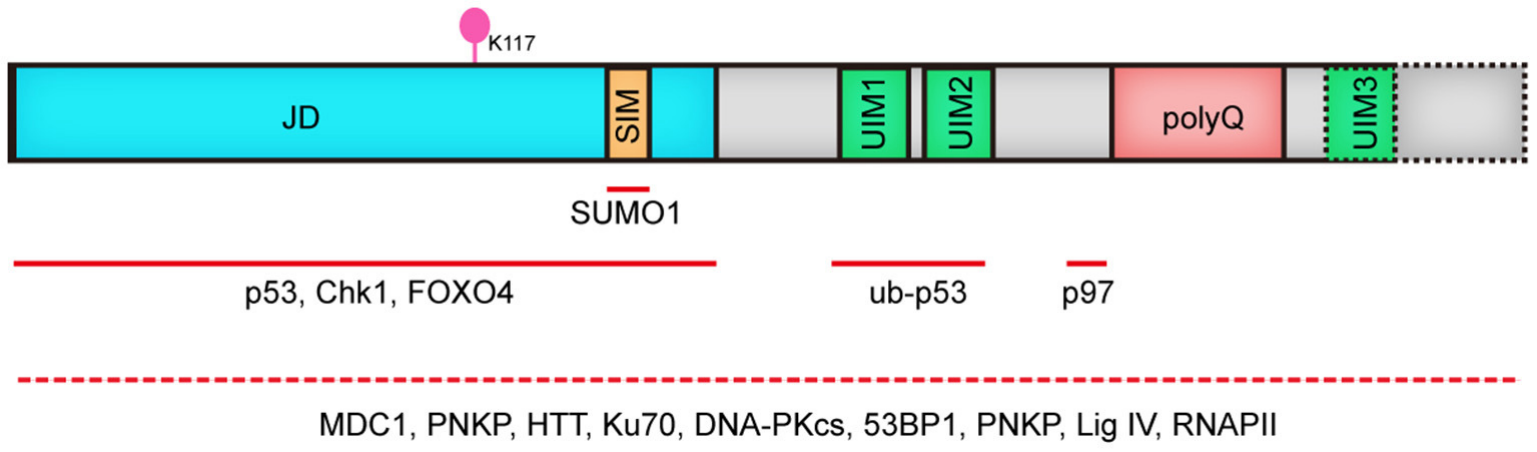
Human ATX3 domain structure. ATX3 is comprised of the catalytic JD, followed by two or three UIMs, (depending on the types of protein isoforms, dashed line illustrates 3UIMs-containing isoform), and the polyQ stretch. JD, josephin domain; SIM, SUMO-interacting motif; UIM, ubiquitin interacting motif; polyQ, polyglutamine; Red lines indicate interacting regions with other proteins. Since the domain mediating the binding of ATX3 to MDC1, PNKP, HTT, Ku70, DNA PKcs, 53BP1, PNKP, Lig IV and RNAPII remains to be elucidated, we used dashed line covering its full length.

*ATXN3* gene was first identified in 1993 and mapped to chromosome 14q24.3-q32.45 by Takiyama and coworkers. It encodes a deubiquitinase (DUB) called MJD1 or ATX3 which contains an unstable CAG repeat (Takiyama et al., 1993; Kawaguchi et al., 1994). The Josephin domain in ATX3, which is highly conserved from yeast to human, confers the deubiquitinase activity, with cysteine14 being the key catalytic residue. Correspondingly, both ATX3 knockout and ATX3 catalytic activity inhibition by mutating Cys14 lead to an obvious increase of polyubiquitinated proteins (Berke et al., 2005; Schmitt et al., 2007), verifying the DUB function of ATX3 *in vivo*. ATX3 also contains 2 to 3 ubiquitin interacting motif (UIM) domains, with the most common isoform found in human brain having 3 UIM domains (Schmidt et al., 1998). UIM domains play an essential role in regulating the DUB activity of ATX3 through their binding to polyubquitinated proteins (Donaldson et al., 2003), determining the cleavage preference to linkage of ubiquitin chain and regulating the ubiquitination state in Josephin domain (Lysine 117 being the primary ubiquitination site) (Berke et al., 2005; Todi et al., 2009, 2010). Collectively, the Josephin domain and UIMs make ATX3 a multifunctional protein that plays vital roles in protein homeostasis, DDR and apoptosis through its interaction with key factors in above-mentioned pathways (Figure 1), and notably, its deubiquitinase activity is indispensable in many cases.

## Functions of ATX3 in DDR

Recent studies unraveled ATX3 to be an essential participant of DDR, including DNA strand break repair, cell cycle arrest, and oxidative stress response. Here, we summarize the key functions of ATX3 in genome integrity maintenance.

### Roles of ATX3 in DNA Strand Break Repair

DNA stand breaks occur as double-strand breaks (DSBs) or single-strand breaks (SSBs). Except in routine cellular processes such as DNA replication, meiosis, and V(D)J recombination, DSBs are generally highly deleterious lesions arising from exposure to ionizing radiation (IR). DSBs can induce chromosomal rearrangement such as deletion, translocation, or amplification. DSBs are repaired primarily by homologous recombination (HR) and non-homologous end joining (NHEJ). The error-free HR repair operates in S and G2 phase, with sister chromatids available as repair templates, while error-prone NHEJ is active throughout the cell cycle. Failure to repair DSBs can trigger permanent growth arrest and finally cell death (Bennett et al., 1993; Sandell and Zakian, 1993). Compared to DSBs, SSBs can be induced by IR and ROS, and repaired by single-strand break repair (SSBR) (Caldecott, 2003; Katyal and McKinnon, 2008).

#### ATX3 Preserves Genome Integrity by Regulating PNKP Phosphatase/Kinase Activity

PNKP is a bi-functional enzyme with 3′-phophotase and 5′-kinase activities, capable of removing 3′-P and phosphorylating 5′-OH, facilitating DNA ligation (Jilani et al., 1999; Weinfeld et al., 2011). The roles of PNKP in DNA strand break repair and BER are particularly important for genome stability of neural cells. Mutations in PNKP (L176F, E326K, T424Gfs48X, and exon15Δfs4X) result in autosomal recessive neurological disorder characterized by microcephaly, seizures and developmental delay (MCSZ) (Shen et al., 2010), and the related cell lines exhibit compromised SSBR following γ-radiation (Ward et al., 1987). Importantly, a lot of evidence suggest that PNKP is also implicated in DSB repair (Chappell et al., 2002; Koch et al., 2004; Karimi-Busheri et al., 2007; Segal-Raz et al., 2011). Although it remains unclear whether the downregulation of kinase activity or phosphatase activity contribute to MCSZ, PNKP mutations cause a significant decrease of PNKP level in MCSZ patients (Shen et al., 2010; Reynolds et al., 2012), indicating the relevance of PNKP abundance.

Accordant with its role in the DDR, PNKP knockdown sensitizes cells to H_2_O_2_ and IR (Rasouli-Nia et al., 2004). PNKP can be phosphorylated at S114 and S126 by ATM (Segal-Raz et al., 2011; Zolner et al., 2011), which prevents its proteasomal degradation by Cul4A-DDB1-STRAP ubiquitin ligase complex and promotes effective DNA repair (Parsons et al., 2012). Moreover, lymphoblast of MCSZ patients displayed a remarkably compromised repair of oxidative DNA damage (Shen et al., 2010).

**Figure 2:**
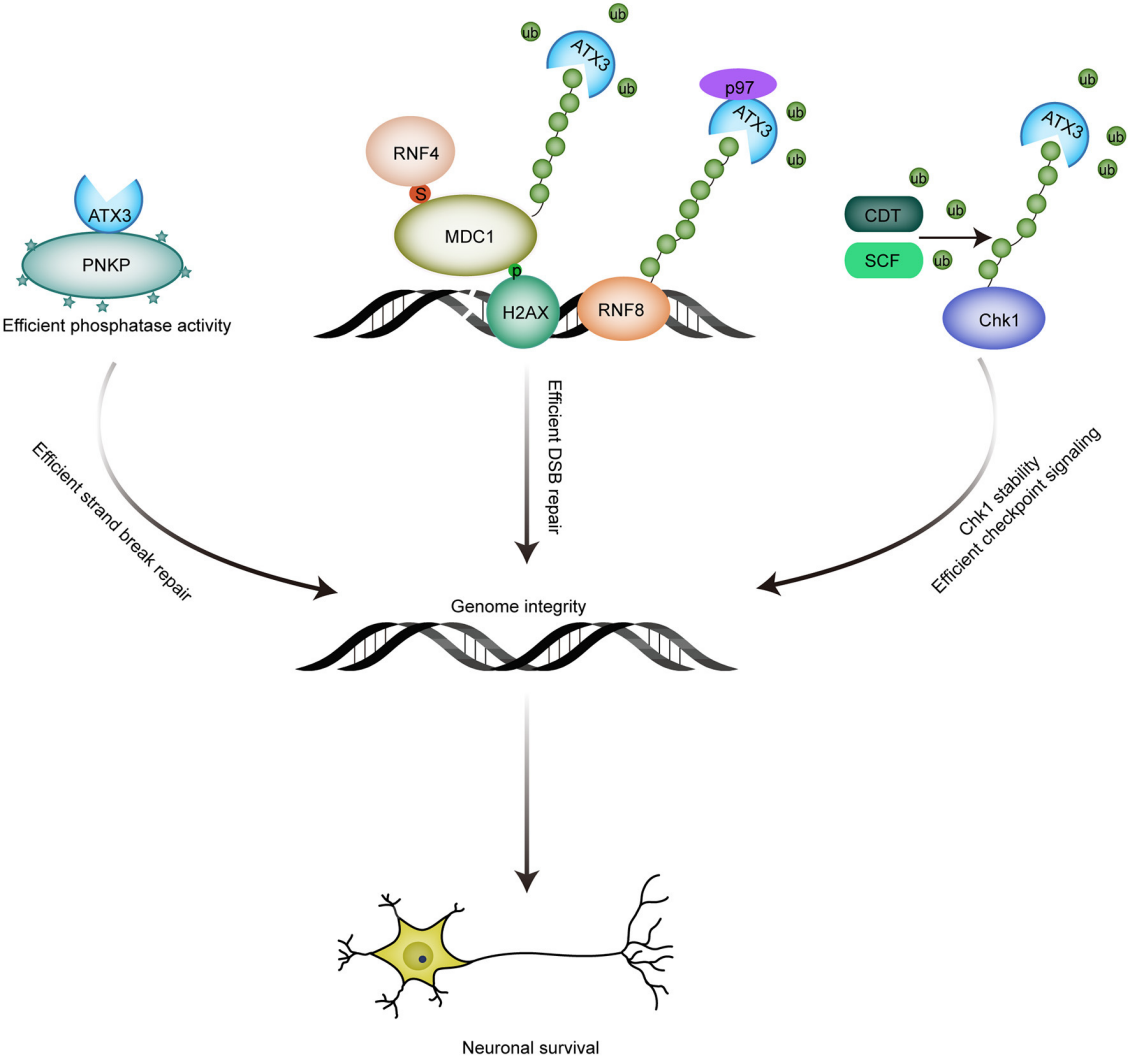
Roles of ATX3 in genome integrity maintenance. (1) The association between ATX3 and PNKP promotes the phosphatase activity of PNKP, and consequent efficient strand break repair; (2) ATX3 antagonizes RNF4-induced polyubiquitination and subsequent chromatin eviction of MDC1, and thus ensures the residence of MDC1 at DSBs sites and efficient DSB repair; p97-ATX3 complex promotes RNF8 stability by antagonizing proteasome-dependent degradation under physiological conditions, while p97-ATX3 complex stimulates RNF8 extraction and ensures proper DSB repair in response to genotoxic stimulus. (3) ATX3 counteracts SCF- and CDT-induced polyubiquitination and degradation of Chk1. By promoting Chk1 stability, ATX3 ensures that checkpoint signaling is accurately activated.

Gao et al. identified that ATX3 co-localizes with PNKP in cells and human brain sections, suggesting an association between ATX3 and PNKP. Accordantly, Chatterjee et al. substantiated the interaction by endogenous immunoprecipitation assays and proximity ligation assay (PLA) (Chatterjee et al., 2015; Gao et al., 2015), and found that this interaction is mediated by both kinase domain and phosphatase domain of PNKP. The association between ATX3 and PNKP is of biological significance to PNKP, as exemplified by the enhanced phosphatase activity of PNKP in an ATX3 dose-dependent manner, although the underlying mechanism remains unclear. Coincidently, ATX3 ablation results in decreased PNKP activity, and sequentially DNA strand breaks accumulation and delayed repair of DNA strand breaks induced by oxidative stress (Chatterjee et al., 2015). All these data demonstrate a role of ATX3 in genome integrity maintenance by promoting phosphatase/kinase activity of PNKP (Figure 2).

#### ATX3 in Classical NHEJ Repair of Transcribed Genes

PNKP was reported to participate in classical NHEJ-mediated error-free repair of DSBs in transcribed genes (Chakraborty et al., 2016). Later, ATX3 was identified to be a component of transcription-coupled DNA repair complex composed of ATX3, HTT, RNA polymerase II subunit A, PNKP, and cyclic AMP-response element-binding (CREB) protein (CBP). This complex senses DNA lesions and promotes their repair during transcriptional elongation (Gao et al., 2019). Given the role of ATX3 in promoting PNKP activity, it raises the possibility that ATX3 might be involved in the PNKP-mediated error-free DSB repair of the transcribed genome. Indeed, ATX3 was recently found to be essential for classical NHEJ repair of DSBs in transcribed genes (Chakraborty et al., 2020). ATX3 interacts with classical NHEJ components (such as Ku70, DNA PKcs, 53BP1, PNKP, and Lig IV), nascent transcripts and RNA polymerase II (RNAP II) under physiological conditions. Analogous to classical NHEJ proteins, ATX3 also exhibits preferential association with transcribed genes, indicating the potential role of ATX3 in classical NHEJ repair. Consistently, ATX3 depletion results in a compromised error-free repair of transcribed genes via classical NHEJ. In addition, ATX3 depletion causes a significant reduction in RNAP II level, probably caused by an enhanced ubiquitination of the stalled elongating RNAP II, which adversely impacts classical NHEJ repair and transcription (Chakraborty et al., 2020). Thus, ATX3 plays an important role in classical NHEJ repair of strand breaks in transcribed genes.

#### ATX3 Reinforces DSB Repair by Promoting the Retention of MDC1

MDC1, best known for its role in cellular response to DSBs, is recruited to the sites of DNA damage by phosphorylated histone variant H2AX (γH2AX), and further facilitates the loading of other DNA damage repair proteins, to promote DNA repair and checkpoint signaling. Previous studies revealed that DNA damage-induced SUMOylation, an ubiquitin-like modifier, recruits the SUMO-targeted ubiquitin ligase (STUBL) RNF4 to the sites of DNA lesions, which promotes the ubiquitin-dependent extraction of MDC1 and RPA, and thus facilitates DSB repair via NHEJ and HR, respectively (Galanty et al., 2012; Luo et al., 2012; Yin et al., 2012). However, RNF4 is recruited to DSBs very quickly at a time when MDC1 removal would be unfavorable for the execution of DNA damage signaling (Vyas et al., 2013; Pfeiffer et al., 2017). Thus, MDC1 must be retained at the sites of DNA lesions to ensure efficient initiation of DNA repair (i.e., the RNF4 activity must be inhibited) at the early stage of DSBs response, and how this could be achieved?

It has been reported that ATX3 can be recruited to DNA damage sites induced by laser microirradiation (Nishi et al., 2014). Recent studies further demonstrated that ATX3 is recruited to DSBs in a SUMOylation-dependent manner. The interaction between ATX3 and SUMO1, mediated by N-terminal Josephin domain and further stimulated by DNA damage, is indispensable for the localization of ATX3 to DSBs (Pfeiffer et al., 2017). The similar spatial-temporal accumulation of ATX3 and RNF4 raises the possibility that they share the same SUMOylated substrates. ATX3 was demonstrated to be responsible for the stable retention of MDC1 at DSBs by repressing the RNF4-dependent ubiquitination of MDC1 (Pfeiffer et al., 2017). The deubiquitinase activity of ATX3 is indispensable for its role in facilitating MDC1 anchoring at DSB sites, which further promotes recruitment of RNF8 and RNF168 and subsequent accumulation of ubiquitination-dependent BRCA1 and 53BP1. Consequently, ATX3 depletion results in impaired DSB repair, as indicated by reduced RPA and RAD51 recruitment, and increased sensitivity to PARP inhibitor. Thus, the deubiquitinase ATX3 prevents premature MDC1 eviction by antagonizing RNF4 to reinforce effective DSB response (Figure 2).

The constitutive interaction between ATX3 and MDC1 is neither stimulated by DNA damage assault nor dependent on the major SUMOylation site of MDC1 (K1840) (Luo et al., 2012), As a SUMO-activated deubiquitinase, ATX3 antagonizes RNF4-mediated MDC1 ubiquitination and its subsequent extraction. Meanwhile, ATX3 depletion also results in compromised recruitment of RPA, which can be SUMOylated and regulated by RNF4 in response to DNA damage (Dou et al., 2010; Galanty et al., 2012; Yin et al., 2012). Whether ATX3 acts on other RNF4 substrates during DDR warrants further investigation.

#### ATX3 Promotes DSB Repair by Stimulating the Extraction of RNF8

RNF8 is an E3 ligase with crucial roles in the ubiquitination of histone H2A and H2AX, and subsequent recruitment of DNA repair factors including BRCA1 and 53BP1 (Huen et al., 2007). Although RNF8 is indispensable for both efficient DNA repair in response to genotoxic stimulus and genome integrity under physiological conditions, the homeostasis of RNF8 was reported to be tightly regulated very recently. Under physiological conditions, RNF8 catalyzes its own K48-linked ubiquitination, which is antagonized by the p97-ATX3 complex, contributing to preservation of RNF8 abundance. Expression of catalytically inactive ATX3-C14A or ubiquitin-binding defective ATX3-UIM^*^ mutants result in accelerated degradation of RNF8, which can be rescued by wild-type ATX3 but not ATX3-VBM (VCP/p97-binding motif) mutant (Boeddrich et al., 2006; Singh et al., 2019), indicating the importance of p97-ATX3 complex in RNF8 stability. Under genotoxic attack, p97-ATX3 complex extracts RNF8 from DNA lesion sites (Singh et al., 2019). Consistently, ablation of either component led to aberrant accumulation of RNF8, defective DNA repair and sensitivity to IR. However, hyperaccumulation of RNF8 observed here is inconsistent with previous finding that RNF8 recruitment is reduced in ATX3 deficient cells. Furthermore, the authors failed to detect the interaction between MDC1 and ATX3 observed by Pfeiffer et al. (2017). Whether this contradiction is caused by different experimental conditions needs further investigation. The homologs of p97 and ATX3 in *C. elegans* was also reported to regulate DSB repair (Ackermann et al., 2016).

### Functions of ATX3 in Cell Cycle Checkpoint

When confronted with DNA damage insults, appropriate cell cycle checkpoint can prevent cells from proceeding to the next cell cycle phase and provide enough time for DNA repair. After completion of DNA repair, the termination of checkpoint signaling allows the resumption of normal cell cycle progression. Failure to repair DNA lesions can lead to permanent cell cycle arrest and apoptosis to the end.

Chk1, activated by various kinds of DNA damage insults including replication stress, interstrand cross-link (ICL) and DSBs, is essential for genome integrity maintenance and cell survival in eukaryotic cells. Chk1 can phosphorylate its downstream effectors to regulate various cellular pathways such as cell cycle checkpoint, DNA repair or cell death if the damage is too severe to be repaired (Takai et al., 2000; Feijoo et al., 2001). Post-translational modifications including phosphorylation and ubiquitination play important roles in modulating Chk1 activity. Recently, chaperone-mediated autophagy (CMA) is also described to play a crucial role in regulating Chk1 activity through degradation of activated Chk1 after genotoxic exposure, promoting checkpoint termination (Park et al., 2015).

After prolonged replication stress, Chk1 can also be targeted for proteasomal degradation by CUL1- and CUL4-containing E3 ligase complexes to terminate checkpoint signaling after completion of DNA repair. However, protecting Chk1 from degradation is absolutely necessary to maintain a steady-state level of Chk1 under unperturbed conditions to ensure proper activation of DNA damage checkpoint and DNA repair signaling in response to DNA damage. Although deubiquitinase USP1 and USP7 are reported to promote Chk1 stability (Guervilly et al., 2011; Alonso-de Vega et al., 2014; Zhang et al., 2014), whether SCF(SKP1-Cul1-FBXO6)- or CDT(Cul4A-DDB1-CDT2)-mediated polyubiquitination of Chk1 can be restrained by deubquitinase(s) remains to be elucidated. Recently, we reported that ATX3 can stabilize Chk1 by antagonizing SCF- and CDT-mediated polyubiquitination and degradation, and ATX3 shows a dynamic regulation on Chk1 stability before and after prolonged replication stress. Under unperturbed conditions and upon DNA damage, ATX3 interacts with Chk1 and protects it from CDT- and SCF-mediated polyubiquitination and degradation, promoting DNA repair and checkpoint signaling. Under prolonged replication stress, ATX3 dissociates from Chk1, concomitant with a stronger association between Chk1 and its E3 ligase, leading to Chk1 degradation and checkpoint termination. Consequently, ATX3 deficiency results in reduced abundance of Chk1, abortive G2/M checkpoint and decreased cell survival after replication stress (Tu et al., 2017). Hence, ATX3 exerts its function in genome integrity partly through stabilizing Chk1 (Figure 2).

### Roles of ATX3 in Oxidative Stress Response

ROS, produced in normal cellular metabolic processes, results in DNA base oxidation and DNA breaks. Due to substantial oxygen consumption of the central nervous system, efficient response to oxidative stress is particularly indispensable for neurons.

The forkhead box O (FOXO) transcription factors are reported to be associated with the regulation of cell cycle arrest, and protection against cell death induced by oxidative stress (Kops et al., 2002; van der Horst and Burgering, 2007). *SOD2* gene, encoding the antioxidant enzyme SOD2 with essential role in removal of ROS, is a well-known target of FOXO family (Kops et al., 2002; van der Horst and Burgering, 2007). ATX3 was reported to interact with the FOXO transcription factor FOXO4 through its Josephine domain and co-activate the FOXO4-dependent transcription of SOD2. Under oxidative stress, nuclear translocation and concomitantly increased binding of ATX3 and FOXO4 to SOD2 promoter can upregulate SOD2. Consistently, ATX3 knockdown leads to a reduced expression of SOD2 (Araujo et al., 2011). Although ATX3 fails to downregulate the ubiquitination level of FOXO4, the increased protein level of FOXO4 by co-expression of ATX3 indicates that ATX3 may participate in the stabilization of FOXO4. It is possible that nuclear localization of ATX3 induced by oxidative stress promotes the stabilization of transcriptionally active FOXO4 and thus cellular response to oxidative stress (Araujo et al., 2011).

ATX3 was also shown to be protective against oxidative stress in a Bcl-xL-dependent manner. ATX3 directly binds to Bcl-xL and promotes the interaction between Bcl-xL and Bax, which cooperate in modulating mitochondrial oxidative stress-induced apoptosis by preventing the activation of Bax (Cheng et al., 1996; Youle and Strasser, 2008; Zhou et al., 2013). Moreover, ATX3 can interact with HHR23 proteins (HHR23A and HHR23B), human homolog of yeast RAD23, which are required for NER (Sugasawa et al., 1997). Thus, it is plausible that ATX3 also participates in NER (Wang et al., 2000).

Above all, ATX3 plays multiple roles in DDR and genome stability. Being post-mitotic cells, neurons must overcome endogenous and exogenous DNA damage sources usually on a lifetime basis. Therefore, the efficient DDR signaling pathway promoted by ATX3 is expected to be crucial for the maintenance of healthy neurons.

## Roles of ATX3 in Apoptosis

Tumor suppressor protein p53 plays a crucial role in modulating cell fate under stress and suppressing the propagation of damaged cells (Muller and Vousden, 2013). As a transcription factor, p53 is involved in various cellular pathways such as DNA repair, cell differentiation, cell cycle progression and apoptosis. To maintain its normal cellular functions, p53 activity must be finely-tuned, and posttranslational modifications, including phosphorylation, acetylation and ubiquitination, play a dominant role in this aspect (Olsson et al., 2007). It is known that E3 ubiquitin ligase MDM2 mediates the ubiquitination of p53 and thus regulating its subcellular localization and degradation. Additionally, E3 ligases such COP1, Pirh2 and ARF-BP1 were also reported to modulate p53 stability or localization (Leng et al., 2003; Dornan et al., 2004; Chen et al., 2005). On the other side, deubiquitination mediated by deubiquitinases provides a parallel important control to p53. OTUB1 from otubain (OTU) family and several deubiquitinases such as USP7 and USP10 from ubiquitin-specific protease (USP) family were suggested to regulate p53 stability and function (Li et al., 2004; Yuan et al., 2010; Sun et al., 2012). Lately, we found that p53 is a substrate of ATX3. Under physiological conditions, Josephin domain of ATX3, DNA-binding domain and the C-terminal regulatory domain of p53 are required for their interaction. During deubiquitination process, ATX3 mainly associates with ubiquitinated p53 through its UIMs domain. ATX3-mediated deubiquitination of p53 promotes the stability of p53, and thus regulates its function in transcription, cell cycle progression and apoptosis. Consistently, ATX3 deletion results in decreased p53 stability, activity and function. Ectopic expression of ATX3 promotes p53-dependent apoptosis in cells and zebrafish (Liu et al., 2016). Therefore, ATX3 functions to facilitate the stability and apoptotic function of p53.

## How POLYQ Expansion Affects the Physiological Functions of ATX3 in DDR and Apoptosis?

SCA3 is considered to be caused by abnormal polyQ expansion in ATX3, although the underlying mechanism remains enigmatic. The age at onset of the disease decreases with increasing polyQ repeats, and there is a positive correlation between the severity of the disease and the length of polyQ tracts (Lee et al., 2015). These facts indicate the relevance of expanded polyQ repeats in SCA3 pathogenesis. One hypothesis is that abnormal polyQ expansion in ATX3 results in protein aggregation, sequestering essential proteins involved in protein quality control and transcription, ultimately provoking cytotoxicity and cell death (Matos et al., 2011). Recent evidences propose the possibility that polyQ expansion of ATX3 might abrogate its functions in DDR and apoptosis, which provides an explanation for the involvement of abnormal DDR and apoptosis in SCA3 pathogenesis.

### The PolyQ Expansion of ATX3 Attenuates DNA Strand Break Repair

**Figure 3:**
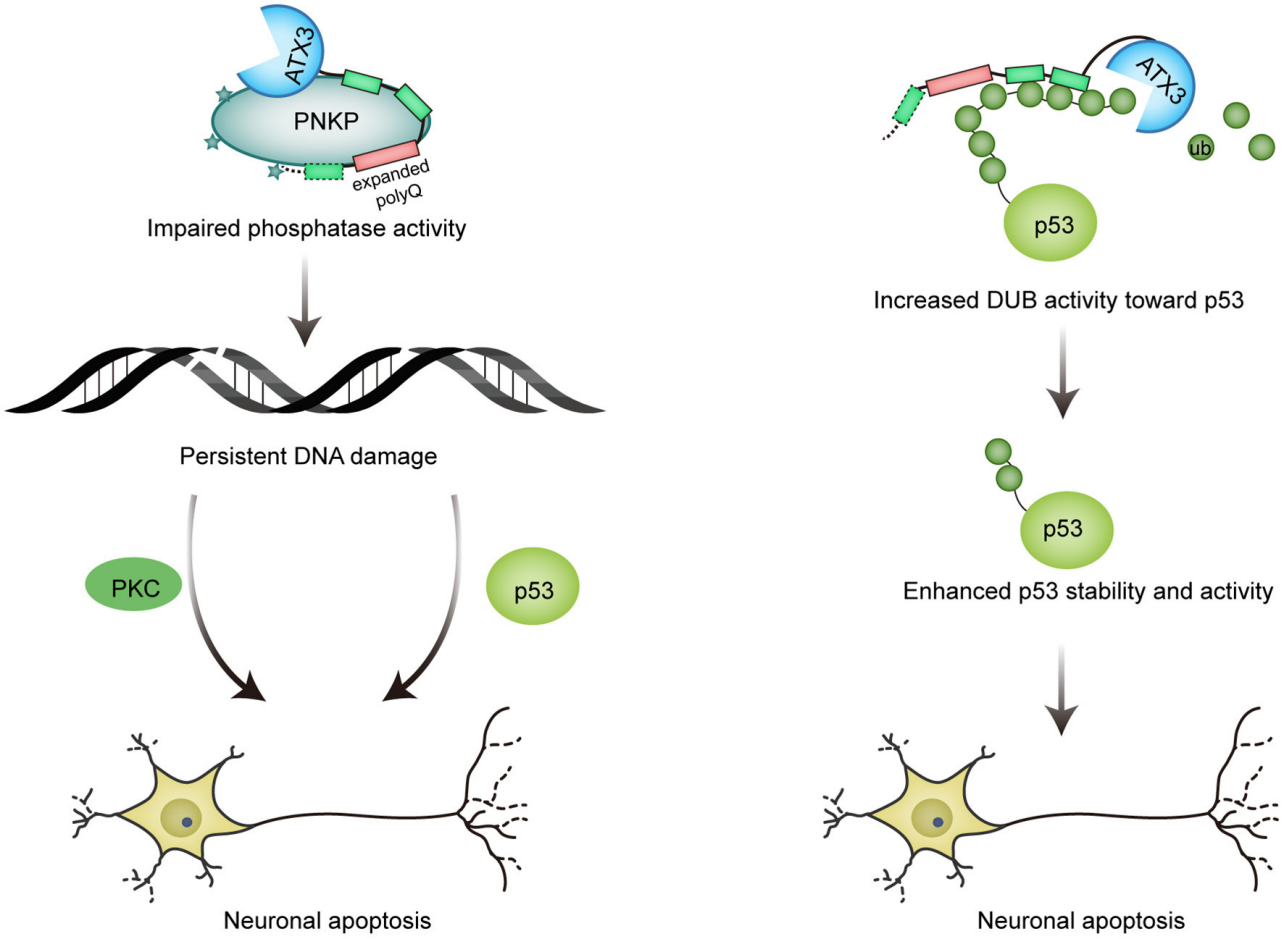
Mutant ATX3 triggers neuronal death. (1) Expanded ATX3 inhibits the phosphatase activity of PNKP, leading to persistent accumulation of DNA damage and prolonged activation of p53 and PKC proapoptotic pathway, which are responsible for neuron loss in SCA3; (2) PolyQ-expanded ATX3 also obtains augmented deubiquitinase activity toward p53, resulting in enhanced stability and activity of p53 and consequent p53-mediated neuronal cell death.

Unlike wild-type ATX3, which promotes PNKP phosphatase activity, the pathological form of ATX3 significantly inhibits PNKP's phosphatase activity *in vitro*. Results from cells and SCA3 mouse also confirm that the expanded ATX3 compromises the activity of PNKP, and that the decreased PNKP activity results in a decreased SSB repair and thus increased DNA damage (Figure 3; Chatterjee et al., 2015).

In accordant with its essential roles in both dividing and postmitotic neurons, PNKP is expressed in both neuronal precursors and differentiated neurons, and PNKP knockdown results in increased apoptosis of neuronal precursors and postmitotic neurons (Shen et al., 2010). Therefore, abnormal polyQ expansion-induced loss of PNKP function is implicated in SCA3 pathogenesis (Gao et al., 2015). PNKP was found to colocalize with both ATX3 and polyQ aggregates in SCA3 brain sections. Additionally, the ectopic expression of expanded-ATX3 promotes the foci formation of 53BP1 and γH2AX, indicating the accumulation of DNA damage. Accumulated DNA damage then promotes p53- and PKC-dependent apoptosis, which triggers neuron death in SCA3 (Shen et al., 2010; Gao et al., 2015). Given that PNKP is recruited to polyQ aggregates, as evidenced by co-localization of PNKP and polyQ aggregates, it is supposed that polyQ-expansion results in sequestration of PNKP, thus abrogating its function in DDR. Recently, it was also reported that phosphatase activity of PNKP is significantly abrogated in affected brain regions of SCA3 mice and SCA3 patients. Consistently, accumulation of DNA damage is observed in SCA3 mice and patients, as revealed by increased level of γH2AX and phosphorylated 53BP1. Furthermore, affected brain regions of SCA3 mice and patients exhibit higher level of strand breaks in transcribed genes. Given the essential role of PNKP in error-free repair of transcribed genes in postmitotic neurons, compromised PNKP activity induced by mutant ATX3 with polyQ expansion might be involved in the pathogenesis of SCA3. In support of that, PNKP overexpression partially rescues SCA3 phenotype in *Drosophila* model of SCA3 (Chakraborty et al., 2020).

The role of ATX3 in counteracting RNF4-induced chromatin removal of MDC1 to consolidate MDC1-dependent DSB response relies on the deubiquitinase activity of ATX3. However, whether the polyQ-expansion affects its deubiquitinase activity is controversial. Some studies support the idea that polyQ expansion has no significant influence on the protease activity of ATX3 (Burnett et al., 2003; Berke et al., 2004), while Winborn and coworkers observed that the expanded ATX3 was less effective in reducing general cellular protein ubiquitination than wild-type ATX3 (Winborn et al., 2008). Whether polyQ expansion of ATX3 affects its deubiquitinase activity toward MDC1 remains to be answered. In addition, expanded ATX3 was reported to retain enhanced interaction with p97 (Zhong and Pittman, 2006). How this enhanced association between p97 and ATX3 affects the DUB activity of ATX3 toward RNF8 and DDR remains elusive.

### Mutant ATX3 Augments Apoptosis

It is thought that polyQ-expanded ATX3 undergoes conformational change and acquires some toxic properties, therefore resulting in altered interactions of ATX3. Liu et al. found that the pathological ATX3 binds to p53 with a stronger affinity compared to wild-type ATX3. Coincidently, mutant ATX3 exhibits stronger deubiquitinase activity toward p53 both *in vitro* and *in vivo*, leading to higher abundance and stability of p53 in mutant ATX3-expressing cells. The activity of p53 is also enhanced by polyQ-expanded ATX3, as evidenced by elevated expression of p53-responsive genes. Further, polyQ expansion of ATX3 causes more severe neurodegeneration in zebrafish and mice in a p53-dependent manner, providing a novel explanation for the pathogenesis of SCA3 (Figure 3; Liu et al., 2016). Interestingly, p53 level is also increased in brains affected by neurodegenerative diseases such as AD, PD and HD (Chang et al., 2012).

Mutant ATX3 expression in primary neuronal cultures derived from cerebellum, striatum, and substantia nigra, which are susceptible to neurotoxicity induced by expanded ATX3 *in vivo*, induces mitochondrial apoptotic death (Chou et al., 2006). More specifically, mutant ATX3 results in upregulation of pro-apoptotic Bax and downregulation of anti-apoptotic Bcl-xL, which leads to mitochondrial release of cytochrome c and Smac followed by activation of caspase-9, an essential initiator of mitochondrial apoptosis (Danial and Korsmeyer, 2004; Green and Kroemer, 2004). Notably, polyQ-expanded HTT also causes similar effects. Thus, polyQ-expanded protein-mediated mitochondrial apoptotic death of affected neurons might be a common mechanism for polyQ diseases (Wang et al., 2003; Choo et al., 2004).

### Compromised Capacity to Activate FOXO4-Mediated SOD2 Expression

Compared with wild-type, polyQ-expanded ATX3 displays impaired ability to activate FOXO-dependent transcription of SOD2. This might result from an increased association of mutant ATX3 to *SOD2* promoter region, the very same region bound by FOXO4, and thus inhibit the FOXO4-mediated SOD2 expression. Accordantly, both mRNA and protein levels of SOD2 are decreased in lymphoblastoid cell (LC) lines and pons tissue from SCA3 patients (Araujo et al., 2011), and SCA3-LCs fails to increase the SOD2 expression in response to oxidative stress. Given the important role of SOD2 in scavenging ROS, impaired SOD2 expression would contribute to increased ROS accumulation in response to oxidative stress. Indeed, ROS level in SCA3 LCs exposed to H_2_O_2_ is obviously increased. Moreover, compared with LCs from unaffected controls, H_2_O_2_ exposure results in significantly decreased cell viability of LCs from SCA3 patients. Considering the rate of oxygen metabolism is relatively high in nervous system, impaired removal of ROS can be particularly detrimental to nervous system. Therefore, reduced expression of SOD2 in SCA3 patients may result in constant accumulation of ROS and cytotoxicity, contributing to neurodegeneration. Consistently, SOD2 knockout mice manifest neurodegeneration and progressive motor disturbance (Lebovitz et al., 1996; Williams et al., 1998).

Besides, a recent work suggests the toxicity of expanded ATX3 in abrogating transcriptional activity of FOXO. The coiled-coil structures of polyQ domain mediate the binding to transcription factor FOXO, and this interaction increases the nuclear localization of FOXO and impairs its transcriptional activity. Consequently, the mRNA levels of its representative target genes which are involved in dendrite morphogenesis or neurogenesis are significantly decreased. Moreover, the coiled-coil structures of expanded ATX3 cause dendrite defects in *Drosophila* dendritic arborization neurons as well as behavior abnormalities (Kwon et al., 2018).

### Selective Tissue Vulnerability in SCA3

Selective neuronal loss and DNA damage accumulation are key features of many neurodegenerative disorders including SCA3 and HD, although the underlying mechanism remains unclear. High level of oxygen consumption in brain results in significantly increased oxidative stress, and postmitotic neurons rely on limited repair pathway such as NHEJ to counteract DNA damage assaults, which contribute to higher sensitivity of the nervous system. Mutant ATX3-mediated compromised DNA repair and increased ROS might be responsible for selective tissue vulnerability. Both WT and mutant ATX3 are shown to express in pontine nuclei, substantia nigra and dentate nuclei, regions mostly affected in SCA3 patients. In contrast, expression level in unaffected regions such as striatum, cortex and hippocampus is much lower (Chen et al., 2008). This differential expression pattern in brain might partially explain the selective tissue vulnerability in SCA3. Additionally, brain extracts from affected cerebellum region in SCA3 patients or affected brainstem (but not unaffected forebrain) in SCA3 mice model, specifically inhibit PNKP activity (Chakraborty et al., 2020). Consistent with the essential role of PNKP-mediated classical NHEJ repair in postmitotic neurons (Chatterjee et al., 2015; Chakraborty et al., 2016), higher level of DNA damage accumulates in affected brain regions of SCA3 patients and mice model (Gao et al., 2019; Chakraborty et al., 2020). Thus, selective PNKP activity impairment, DNA damage accumulation, and neuronal loss might represent potential biomarkers for SCA3 and HD.

## Prospects for Therapy of SCA3 and Other Neurodegenerative Diseases

Age-related neurodegenerative disorders including SCA3 and HD show elevated DNA damage (Illuzzi et al., 2009; Chatterjee et al., 2015), and significant advances have been achieved about the relevant molecular pathways. The role of aberrant apoptosis in the pathogenesis of these diseases are also reported (Ghavami et al., 2014; Liu et al., 2016). These studies might provide novel guideline for development of effective therapy of these neurodegenerative disorders. Here, we summarized preclinical studies targeting DDR and related apoptosis for treatment of neurodegenerative diseases, focusing on polyQ disorders including SCA3 and HD.

### DNA Damage Response Pathways as Potential Therapeutic Targets

Decades of research in genetics and molecular biology have established the connection between inherited DNA repair defects and progressive neurodegenerative diseases, such as xeroderma pigmentosum, Cockayne syndrome, ataxia telangiectasia, and among many others (Jeppesen et al., 2011; Maiuri et al., 2017). Dysfunctional DDR are also implicated in several neurodegenerative polyQ diseases such as SCA3 and HD. A genome-wide association analysis found that genes involved in mismatch repair can modulate HD's age of onset (Lee et al., 2015). Other studies also indicate that DNA repair enzymes significantly modify the onset age in polyQ disorders including SCAs and HD (Bettencourt et al., 2016; Moss et al., 2017). DNA damage and apoptosis have also been linked to spinocerebellar ataxia with axonal neuropathy (SCAN1) pathogenesis (Takashima et al., 2002). Mutant AT1 and mutant HTT, responsible for SCA1 and HD, respectively, are shown to reduce the level of HMGB1/2, involved in regulating transcription and DNA repair (Muller et al., 2001; Travers, 2003). And HMGB1/2 complementation ameliorates mutant protein-induced pathology in neurons and in *Drosophila* model (Qi et al., 2007). Another systematic analysis of SCA1 *Drosophila* reveals the roles of aberrant DNA damage repair in SCA1 (Barclay et al., 2014). In addition, spinal and bulbar muscular atrophy (SBMA), another polyQ disorder, is caused by polyQ expansion in androgen receptor (AR). Mutant AR inhibits recruitment of PITP, a DNA repair protein, to damage sites, resulting in sensitivity to DNA damage and genome instability. And a higher level of DNA damage and activation of DDR are observed in SBMA mice (Xiao et al., 2012). Abnormal DDR might be a common underlying molecular mechanism in these neurodegenerative disease, and represent a potential therapeutic target.

#### ATM

Ataxia-telangiectasia mutated (ATM) is a central kinase of DNA damage response. Phosphorylated H2AX mediated by ATM serves as a platform to recruit other DNA damage repair proteins (Ciccia and Elledge, 2010). Aberrant ATM pathway exerts important role in the onset of SCA3: mutant but not wild-type ATX3 expression in SH-SY5Y cells remarkably activates ATM signaling pathway, as indicated by phosphorylation of ATM and its downstream targets including H2AX, Chk2 and p53, and phosphorylation of p53 and Chk2 is diminished by ATM inhibitor KU-55933, suggesting that DDR evoked by mutant ATX3 relies on ATM. Moreover, mutant ATX3 activates pro-apoptotic pathway by activating ATM, which can be restored by ATM inhibition (Gao et al., 2015). And ectopic expression of mutant HTT results in increased ROS levels, DNA damage and ATM activation (Giuliano et al., 2003; Illuzzi et al., 2009; Bertoni et al., 2011). Elevated ATM signaling and γH2AX levels was also observed in cells from HD patients and HD mouse (Giuliano et al., 2003; Enokido et al., 2010). These results imply that inhibition of ATM signaling might be protective against genotoxicity induced by mutant HTT. Indeed, ATM reduction alleviates motor deficits caused by mutant HTT in *drosophila* and mouse models, and pharmacological inhibition of ATM by KU-60019 or KU-55933 also exerts neuroprotective effects in rat striatal neurons and HD patient iPSC-derived neurons (Lu et al., 2014). Hence, ATM might be a potential therapeutic target for SCA3 and HD.

#### PNKP

Recently, both mutant ATX3 and mutant HTT were reported to impair PNKP activity, disrupting strand break repair and transcription. Consistently, there is a higher level of DNA breaks in transcribed genes in affected brain regions of SCA3 patients and mice, and brains of HD patients and mice also show DNA damage accumulation as compared to control (Gao et al., 2019; Chakraborty et al., 2020). In addition, mutant HTT impairs ATX3 activity, which promotes ubiquitination and degradation of CBP, negatively impacting transcription (Gao et al., 2019). More importantly, upregulation of PNKP activity rescues deficient DSB repair and neurotoxicity in SCA3 *Drosophila*, and PNKP overexpression significantly rescues cell toxicity induced by mutant HTT (Gao et al., 2019; Chakraborty et al., 2020). These studies indicate the potential of PNKP as a therapeutic target for SCA3 and HD.

In addition, elevated ADP-ribose levels after DNA damage are observed in PNKP-patient cells (Hoch et al., 2017). Given that mutant ATX3 can inhibit phosphatase activity of PNKP and result in accumulation of DNA breaks (Gao et al., 2015), it might also cause PARP1 hyperactivation. To figure out the therapeutic potential of PAPR inhibition in SCA3 therapy, further studies are still needed.

### Apoptosis as Therapy Targets

Apoptosis is regarded as the dominant mechanism underlying neurodegeneration in PD (Kountouras et al., 2012). A lot of studies also support the role of apoptosis in SCA3 and HD (Sawa et al., 1999; Wellington et al., 2000; Vis et al., 2005). Tumor suppressor protein p53 is known to play a crucial role in deciding cell fate under stress and suppressing the propagation of damaged cells. As mentioned above, mutant ATX3 causes neurodegeneration via apoptosis by upregulating p53 function, and neurodegeneration in animal (zebra fish and mouse) models is remarkably halted by p53 deficiency (Liu et al., 2016). Besides, mutant ATX3 activates apoptosis pathway mediated by p53 and PKC after prolonged accumulation of DNA damage (Gao et al., 2015). Thus, inhibiting p53 activity in neurons could be a potential therapeutic strategy for SCA3.

It was previously reported that p53 is involved in the pathogenesis of HD (Steffan et al., 2000; Trettel et al., 2000) and ployQ-expanded HTT results in transcription dysregulation by interacting with transcription factors such as p53 (Yu et al., 2002; Schaffar et al., 2004; Bae et al., 2005; Cong et al., 2005). There are results indicating that p53 expression level is elevated in HD patients and mice, and that polyQ-induced toxicity is mediated by p53. Consistently, p53 perturbation by pharmacologic inhibitor pifithrin-α (PFT-α), RNAi or genetic deletion significantly rescue the neurodegeneration in HD models (Bae et al., 2005). Similarly, p53 inhibitor PFT-α and p53 knockdown can efficiently relieve polyQ-induced neuronal cell death (Anne et al., 2007). Moreover, PFT-α can enhance the survival of dopamine cell transplants and augment behavioral recovery in parkinsonian animals, which indicates that p53 may be also served as a potential therapeutic target for HD and PD (Chou et al., 2011).

## Conclusion

Although significant advances have been made in establishing normal functions of ATX3 and etiology of SCA3, the underlying molecular mechanism of SCA3 pathogenesis is still urging for sussing out. The impaired functions of ATX3-interacting proteins, sequestered in the polyQ aggregates, is thought to associate with cellular toxicity and neurodegeneration in SCA3 (Paulson et al., 1997; Warrick et al., 1998; Chai et al., 1999a,b, 2002; Ferrigno and Silver, 2000; McCampbell et al., 2000; Schmidt et al., 2002). In the present context, we have tried to give a comprehensive overview of the novel physiological functions of ATX3 in DDR and apoptosis, which relies on interaction between ATX3 and key players in these pathways.

Given that the post-mitotic status of neurons and high level of oxygen metabolism in brain, efficient DDR is absolutely essential for neuronal function and survival. ROS are reported to be responsible for various neurological disorders such as AD and PD. Although the underlying mechanism that contributes to selective neuronal death and pathological changes remains to be investigated, it is thought that the role of ROS in the pathogenesis of these disorders is associated with proteins including a-synuclein, DJ-1, Amyloid β and tau protein (Jiang et al., 2016). For example, oxidative stress can result in upregulated expression of β-secretase and aberrant phosphorylation of tau (Lovell et al., 2004; Tamagno et al., 2005), and oxidative stress-induced damage may compromise the functions of crucial molecules such as Parkin. In comparison with these oxidative stress-induced changes of essential proteins and consequent pathology of AD or PD, ATX3 is directly involved in counteracting oxidative stress by enhancing the association between Bcl-xL and Bax or promoting the expression of antioxidant SOD2 (Araujo et al., 2011; Zhou et al., 2013). More particularly, ATX3 itself is involved in DNA strand break repair by stimulating the activity of PNKP and maintaining the accumulation of MDC1 at breaks, which are both essential for the removal of oxidative stress-induced DNA lesions. Importantly, aberrant polyQ expansion, which is defined as the cause of SCA3, compromises the crucial roles of ATX3 in counteracting oxidative stress and maintaining genome integrity, resulting in neuronal dysfunction and cell death. Current findings about the direct roles of ATX3 and HTT in DDR and apoptosis, and abnormal of which caused by polyQ expansion will help us decipher the molecular pathogenic mechanism of SCA3 and HD. Discussing the molecular changes and related pathways shared by these neurodegenerative diseases would lead to a better understanding of the network in these disorders and facilitating to develop therapeutic strategy for these disorders. Consistently, there are many studies indicating that targeted modulation of DDR and apoptosis can relieve the pathology of many neurodegenerative diseases including HD and SCA3 (Lu et al., 2014; Gao et al., 2015, 2019; Chakraborty et al., 2020).

DDR is also an important pathway in cancer genesis. Sharing the same pathway, it seemed that neurodegeneration and cancer have a subtle linkage, and this relationship alerts us that we need to cautiously assess the cancer risk if we plan to use drugs targeting DDR to slow down the progress of neurodegenerative diseases. For example, p53 is abnormally activated in SCA3, inhibiting the function of p53 may alleviate the degeneration disease, whereas bring about high risk of cancer. Therefore, although many preclinical studies indicate the efficiency of DDR and apoptosis modulation in improving the pathology of neurodegenerative diseases including SCA3, further investigation is undoubtedly needed to confirm target specificity and minimize side effects before their employment for therapeutic intervention. We hope that future studies regarding SCA3 and neurodegenerative diseases will provide effective solutions for clinical therapy.

## Author Contributions

All authors listed have made a substantial, direct and intellectual contribution to the work, and approved it for publication.

## Conflict of Interest

The authors declare that the research was conducted in the absence of any commercial or financial relationships that could be construed as a potential conflict of interest.
